# Facile fabrication of Cu-based alloy nanoparticles encapsulated within hollow octahedral N-doped porous carbon for selective oxidation of hydrocarbons†
†Electronic supplementary information (ESI) available. See DOI: 10.1039/c8sc03531h


**DOI:** 10.1039/c8sc03531h

**Published:** 2018-09-18

**Authors:** Hong Zhong, Yangxin Wang, Caiyan Cui, Feng Zhou, Shuangqi Hu, Ruihu Wang

**Affiliations:** a State Key Laboratory of Structural Chemistry , Fujian Institute of Research on the Structure of Matter , Chinese Academy of Sciences , Fuzhou , Fujian 350002 , China . Email: ruihu@fjirsm.ac.cn; b School of Environmental and Safety Engineering , North University of China , Taiyuan 030051 , China

## Abstract

Hollow carbon materials with versatile chemical compositions and complicated shell architectures hold great promise in heterogeneous catalysis. However, it is a daunting challenge to synthesize metal alloy nanoparticles (NPs) supported by hollow nanostructures. Herein, we present a simple approach for facile fabrication of Pd–Cu alloy NPs embedded in hollow octahedral N-doped porous carbon (Pd–Cu@HO-NPC). The hollow material is derived from HKUST-1 coated by an imidazolium-based ionic polymer (ImIP). Water-sensitive HKUST-1 is simultaneously removed in the process of anion exchange between bromide in the ImIP shell and tetrachloropalladate in aqueous medium. The released Cu(ii) ions and exchanged Pd(ii) ions serve as Cu and Pd sources in the subsequent pyrolysis. The resultant Pd–Cu@HO-NPC exhibits high catalytic activity, selectivity, stability and recyclability in the aerobic oxidation of hydrocarbons. More attractively, the synthetic strategy is of excellent generality, and could be extended to the synthesis of Cu-based bimetallic and trimetallic alloy NPs, such as Pt–Cu@HO-NPC and Pd–Pt–Cu@HO-NPC. This work highlights the superiority of water-sensitive metal–organic frameworks in the ingenious design of hollow carbon materials incorporated with well-dispersed metal alloy NPs.

## Abstract 2

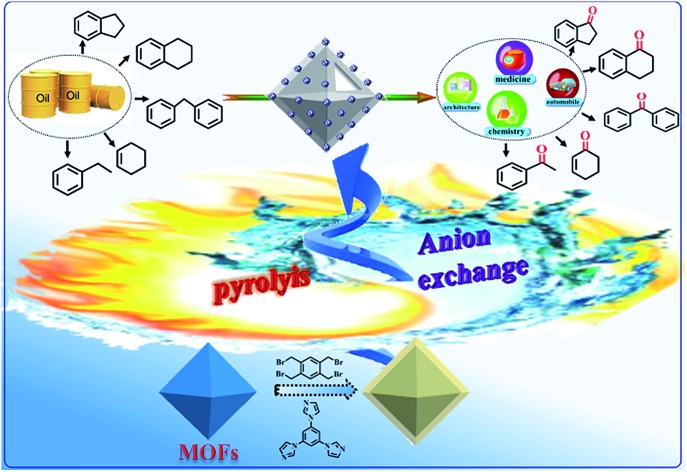
HKUST-1 serves as a template for an imidazolium-based ionic polymer; anion exchange and subsequent topotactic transformation generate hollow nitrogen-doped porous carbon incorporated with Cu-based alloy nanoparticles.

## Introduction

Supported metal nanocatalysts have captured increasing interest in fine chemical industry, environmental protection and energy conversion.^1^–^4^ Hollow carbon materials with large internal voids and porous shells are one type of promising support of metal nanoparticles (NPs).^5^–^8^ The shell could function as a barrier to prevent coalescence of the embedded metal NPs, and the pores in the shell could provide a highway for free diffusion of substrates to access metal active sites.^5^,^9^–^11^ Considerable progress has been achieved in the preparation of hollow porous materials,^12^–^14^ and the application of several hollow nitrogen-doped carbon materials incorporated with noble metal NPs has also been explored in heterogeneous catalysis.^6^,^15^ Alloying a parent noble metal with a non-precious metal holds great promise to improve the catalytic performance and minimize the usage of noble metals,^16^,^17^ but metal alloy NPs supported by hollow carbon materials have not been reported owing to the complicated fabrication processes with harsh reaction conditions and/or the use of expensive additives. It is highly desirable to develop a simple yet effective strategy to integrate metal alloy NPs into hollow heteroatom-doped carbon materials for the design of advanced nanocatalysts.

Templating methods are considered as straightforward and versatile approaches for the synthesis of hollow carbon nanostructures.^18^–^20^ The compatibility between the template surface and carbon precursors has a pivotal influence on the homogeneity of carbon shells and the dispersion of metal NPs.^5^ The deliberate selection and modification of carbon precursors play crucial roles in determining the final physical and chemical properties of the resulting carbon shells.^21^ In this context, main-chain imidazolium-based ionic polymers (ImIPs) are one type of promising precursor to synthesize heteroatom-doped carbon materials. The counter halide anions could facilely exchange with various heteroatom-containing and/or metal-containing anions,^22^–^24^ which provides great opportunities to control the loading amount and the types of metal precursors in ImIPs. Metal and/or metal alloy NPs could be expected after subsequent pyrolysis without the extra addition of other heteroatoms and metal ions. Importantly, ImIPs could serve as robust physical barriers to effectively resist the sintering of metal NPs during pyrolysis, resulting in uniform distribution and high dispersion of metal NPs. The incorporated heteroatoms in the carbon lattice not only modify their chemical and electrical properties, but also enhance the interactions between the carbon support and the embedded metal NPs to trigger superior catalytic performance.^14^ Despite these attractive advantages, as far as we are aware, there is no report on ImIP-based hollow materials. One of the main reasons for this is that the flexible backbone of ImIPs is liable to deformation and even collapse after the removal of templates. It is a daunting challenge to construct hollow ImIPs for application as the precursors of supported metal nanocatalysts.

Metal–organic frameworks (MOFs), assembled by metal ions/clusters and organic ligands, are suitable templates for the construction of hollow structures owing to their facile synthesis, fascinating morphologies, various compositions and tuneable particle sizes.^25^,^26^ Although MOFs have been extensively used as sacrificial templates to fabricate hollow metal and/or metal oxide composites,^27^–^30^ hollow carbon materials derived from MOF templates have rarely been reported, and more efforts are needed to explore the advantages of MOF templates in the synthesis of hollow carbon materials with versatile chemical compositions and complicated shell architectures. It is well known that most of the MOFs are unstable in water; the instability provides tremendous opportunity for disassembly of MOF cores in MOF@ImIP core–shell structures during anionic exchange of ImIPs under mild conditions, requiring no additional procedures for the removal of MOF templates.^31^,^32^ The released metal ions and organic ligands could be located in the pores of the hollow shell, which not only sustains the original shape of ImIP shells, but also serves as the precursor of carbon and/or metal NPs. After the carbonization, the hollow ImIPs containing modifiable metal ions and heteroatoms could be topotactically transformed into heteroatom-doped porous carbon materials containing homogeneously embedded metal alloy NPs, which brings out unique chemical and physical properties that are not attainable from single MOF or ImIP precursors.

As a proof-of-concept study, herein, we developed a facile and versatile method for the synthesis of well-dispersed M–Cu alloy NPs embedded in hollow octahedral nitrogen-doped porous carbon (M–Cu@HO-NPC, M = Pd, Pt and Pd–Pt) using water-sensitive HKUST-1 as a template. They were readily prepared through topotactic transformation of hollow M–Cu@HO-ImIPs, which were obtained through anion exchange of core–shell-structured HKUST-1@ImIP with metal-containing anions in water. To the best of our knowledge, this is the first report on metal alloy NPs supported by hollow nitrogen-doped carbon materials. Pd–Cu@HO-NPC exhibits high catalytic activity, selectivity and recyclability in the oxidation of hydrocarbons using air as an oxidant in the absence of co-catalysts and additives.

## Results and discussion

**Scheme 1:**
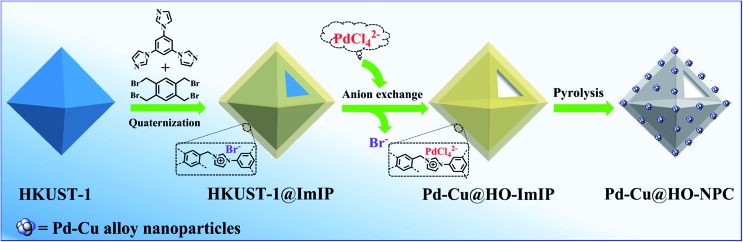
Schematic illustration of the synthesis of Pd–Cu@HO-NPC.

HKUST-1 has been widely employed as a self-sacrificial template in the preparation of metal oxide composites,^33^,^34^ but its application in the synthesis of hollow carbon materials has not been reported so far. As shown in Scheme 1, HKUST-1 was prepared through the assembly of 1,3,5-benzenetricarboxylic acid (H_3_BTC) and Cu(NO_3_)_2_·3H_2_O.^35^ ImIP was readily coated on the surface of HKUST-1 through the quaternization reaction of 1,2,4,5-tetrakis(bromomethyl)benzene and 1,3,5-tri(1*H*-imidazol-1-yl)benzene to give rise to an HKUST-1@ImIP core–shell material; subsequent anion exchange between bromide in the ImIP shell and tetrachloropalladate generated hollow Pd–Cu@HO-ImIP, in which the HKUST-1 template was decomposed simultaneously during anion exchange, and no tedious steps or hazardous reagents were employed for the removal of templates when compared with conventional hard-template methods.^5^,^6^,^12^

**Fig. 1:**
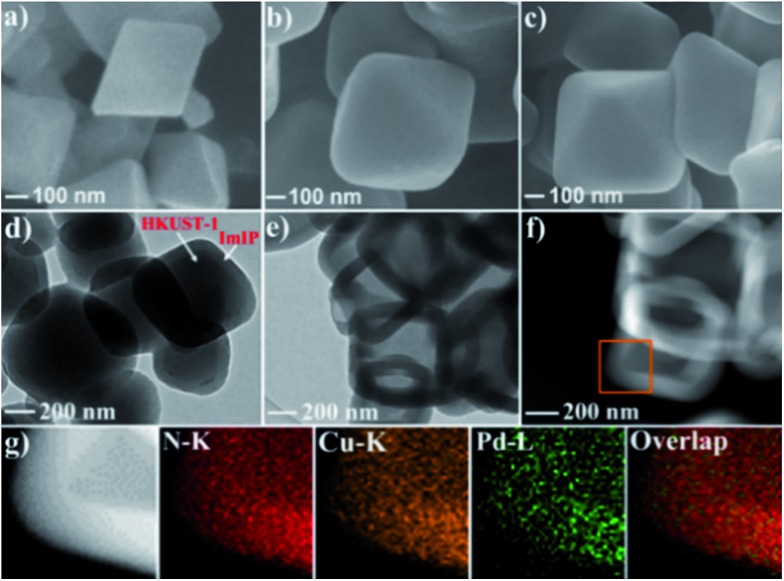
SEM images of (a) HKUST-1, (b) HKUST-1@ImIP and (c) Pd–Cu@HO-ImIP. (d) TEM image of HKUST-1@ImIP. (e) TEM image, (f) HAADF-STEM image and (g) EDX mapping images of Pd–Cu@HO-ImIP.

Scanning electron microscopy (SEM) images reveal that HKUST-1 is octahedron-shaped nanocrystals with the edge lengths in the range from 200 to 400 nm (Fig. 1a). After coating with ImIP, the octahedral morphology is retained in HKUST-1@ImIP, but the edge lengths are increased to 300–600 nm (Fig. 1b). Thus, the thickness of the ImIP shell in HKUST-1@ImIP is in the range of 50–100 nm. Notably, the morphology and size of Pd–Cu@HO-ImIP are almost identical to those of HKUST-1@ImIP, indicating that the shell structure is well maintained after the decomposition of HKUST-1 (Fig. 1c). Transmission electron microscopy (TEM) images show that HKUST-1@ImIP possesses an octahedral core–shell structure (Fig. 1d). ImIP is homogenously coated on the surface of HKUST-1 nanocrystals with a thickness of 50–100 nm, which is consistent with the SEM results. Interestingly, Pd–Cu@HO-ImIP exhibits a hollow octahedral structure (Fig. 1e), and no HKUST-1 residues are observed. The shape and size of the inner cavity match well with those of the original HKUST-1 nanocrystals, and no structural collapse and/or deformation are observed in the external shells after the removal of the kernel template. To the best of our knowledge, this is the first report on hollow ionic polymers with flexible backbones. The outstanding stability of hollow Pd–Cu@HO-ImIP is probably attributed to the assistance of released metal ions and organic ligands from HKUST-1, and they reside in the pores of the shell through electrostatic and coordination interactions to sustain the original shape of the hollow ImIP shells.^22^,^36^ Notably, the hollow octahedral structure was dramatically squashed when Pd–Cu@HO-ImIP was washed with aqueous EDTA-2Na solution to remove metal ions (Fig. S1†). The high-angle annular dark-field scanning TEM (HAADF-STEM) images further confirm the hollow octahedral structure of Pd–Cu@HO-ImIP (Fig. 1f). The corresponding elemental mapping images show that Cu (orange) and Pd (green) co-exist in the octahedral shell (Fig. 1g); they are derived from exchanged tetrachloropalladate ions and released Cu^2+^ ions in HKUST-1, respectively. The distributions of Pd and Cu are identical to each other, which is conducive to *in situ* growth of Pd–Cu alloy NPs during subsequent pyrolysis.^22^

**Fig. 2:**
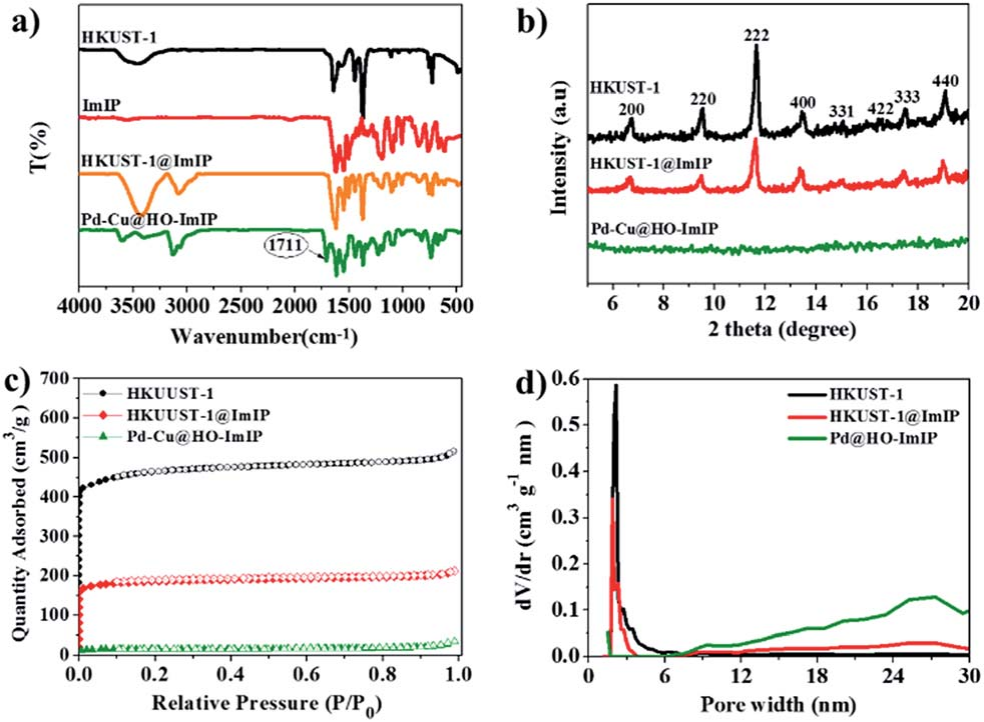
(a) FTIR spectra, (b) XRD patterns, (c) N_2_ adsorption/desorption isotherms and (d) pore size distribution of HKUST-1, HKUST-1@ImIP and Pd–Cu@HO-ImIP.

In the Fourier-transform infrared (FTIR) spectrum of HKUST-1@ImIP, the simultaneous appearance of the characteristic peaks of HKUST-1 and ImIP reveals their successful combination in the core–shell material (Fig. 2a). An extra peak at 1711 cm^–1^, which corresponds to the carboxylate group of BTC,^35^ appears in the FTIR spectrum of Pd–Cu@HO-ImIP, indicating the decomposition of HKUST-1 and the incorporation of the released BTC into the polymer shell, which serves as an adsorption site of Cu(ii). Thermogravimetric analysis (TGA) curves show that HKUST-1@ImIP possesses a lower thermal stability than HKUST-1 owing to the presence of the ImIP shell, and Pd–Cu@HO-ImIP begins to decompose after 200 °C (Fig. S2†).^22^ Powder X-ray diffraction (XRD) patterns indicate that the crystallinity of HKUST-1 is well retained in HKUST-1@ImIP (Fig. 2b),^35^ and no additional diffraction peaks from the ImIP shell are detected. However, the characteristic diffraction peaks of HKUST-1 totally disappear in the XRD pattern of Pd–Cu@HO-ImIP, which further reveals that the HKUST-1 octahedral nanocrystals decompose after anion exchange.

The porous properties of HKUST-1, HKUST-1@ImIP and Pd–Cu@HO-ImIP were investigated by N_2_ adsorption/desorption at 77 K (Fig. 2c). A rapid nitrogen uptake at very low relative pressure (*P*/*P*_0_ < 0.01) in HKUST-1 and HKUST-1@ImIP indicates the presence of extensive micropores,^37^ while the nitrogen uptake in Pd–Cu@HO-ImIP is negligible. The BET surface areas of HKUST-1 and HKUST-1@ImIP are 1776 and 816 m^2^ g^–1^, respectively. As expected, the BET surface area of Pd–Cu@HO-ImIP is as low as 37 m^2^ g^–1^ owing to both the flexibility of the ImIP shell and the pore-filling by metal ions and counter anions.^22^ The pore size distribution reveals that the predominant pores in HKUST-1 and HKUST-1@ImIP are micropores (Fig. 2d), while the pores in Pd–Cu@HO-ImIP are in the range of mesopores, which is consistent with the results of their nitrogen adsorption–desorption isotherms.

**Fig. 3:**
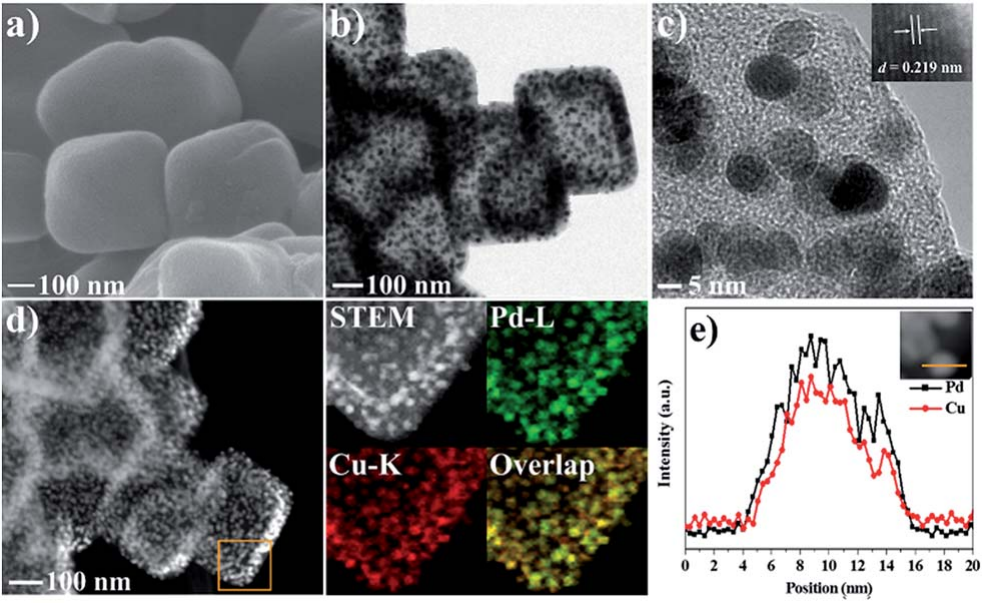
(a) SEM image, (b and c) TEM images, (d) HAADF-STEM and EDX mapping images, and (e) line-scan analysis results of Pd–Cu@HO-NPC.

Considering the unique hollow octahedral structure, high concentrations of carbon and nitrogen species and homogeneous distribution of Pd and Cu in Pd–Cu@HO-ImIP, its topotactic transformation at 500 °C under an inert atmosphere gave rise to hollow octahedral nitrogen-doped porous carbon incorporated with Pd–Cu alloy NPs (Pd–Cu@HO-NPC). SEM and TEM analyses indicate that Pd–Cu@HO-NPC possesses a similar hollow octahedral morphology to Pd–Cu@HO-ImIP (Fig. 3), but the edge lengths and the shell thickness are decreased to 250–500 nm and 30–60 nm, respectively, which is probably attributed to volume shrinkage during pyrolysis.^38^,^39^ The HAADF-STEM images of Pd–Cu@HO-NPC further confirm the maintenance of the hollow structure with an octahedral cavity (Fig. 3d). The metal NPs in the carbon shells are ascribed to the Pd–Cu alloy formed by the co-reduction of Pd and Cu cations during pyrolysis. The average size of well-dispersed Pd–Cu alloy NPs is 8.2 ± 0.8 nm (Fig. S3†), which is comparable with that of metal NPs prepared through the pyrolysis of carbon precursors without using extra reductive agents.^6^,^40^ Notably, Pd–Cu alloy NPs are uniformly embedded in the porous carbon shell, and no metal NPs are observed on the exterior surface of the carbon shell and/or in the hollow cavity, which is conducive to suppressing the leaching and agglomeration of active species in the catalytic reactions.^6^ The corresponding elemental mapping images show that the distributions of Pd and Cu are identical to each other, revealing the alloying state of bimetallic Pd–Cu NPs, which is further demonstrated by their line-scan analyses (Fig. 3e). The high-resolution TEM image shows that the intervals between adjacent lattice fringes are 0.219 nm (Fig. 3c), which corresponds to the (111) lattice plane of the Pd–Cu alloy.^41^ In the XRD pattern of Pd–Cu@HO-NPC (Fig. S4†), the diffraction peaks at 41.1, 48.2, 69.7 and 83.9 can be indexed to the (111), (200), (220) and (311) facets of the face-centered cubic (fcc) Pd–Cu alloy, respectively.^42^,^43^ Inductively coupled plasma (ICP) analyses show that Cu and Pd contents are 0.83 and 0.39 mmol g^–1^, respectively. The N content in Pd–Cu@HO-NC is 5.8 mmol g^–1^ as determined by elemental analysis.

**Fig. 4:**
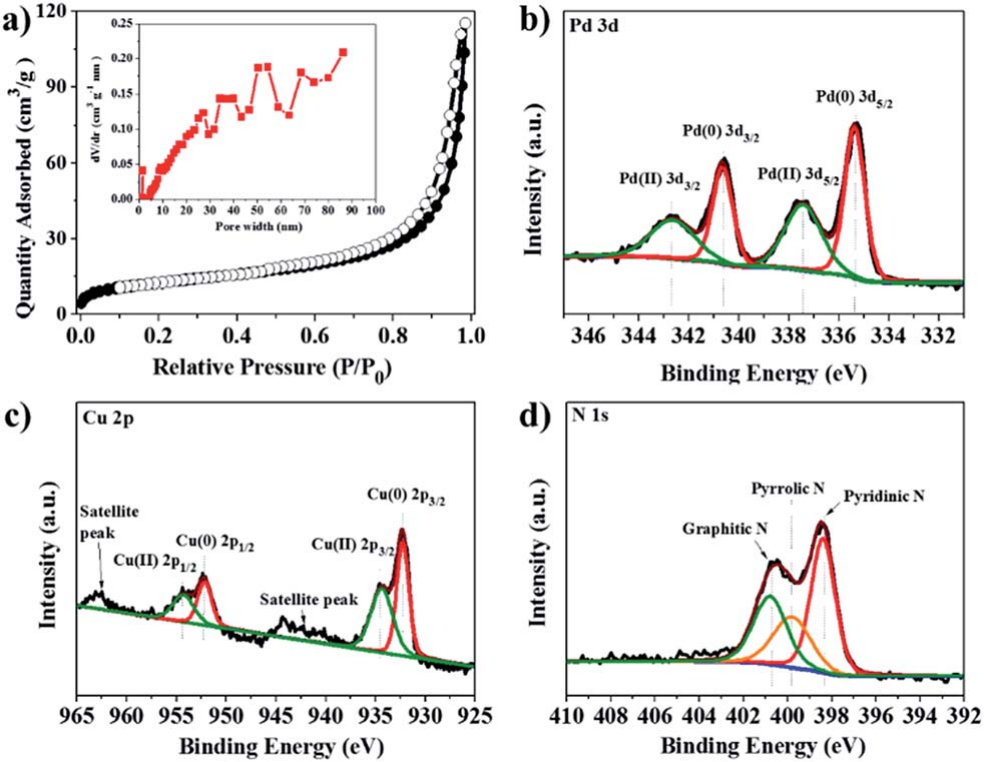
(a) N_2_ adsorption/desorption isotherms and pore size distribution (inset), and (b) Pd 3d, (c) Cu 2p and (d) N 1s XPS spectra of Pd–Cu@HO-NPC.

The N_2_ adsorption/desorption isotherms and relevant pore size distribution show that Pd–Cu@HO-NPC predominantly possesses mesopores (Fig. 4a); the BET surface area and total pore volume are 121 m^2^ g^–1^ and 0.21 cm^3^ g^–1^, respectively. The combination of the mesopores with the large hollow cavity generated by the self-sacrificial HKUST-1 is favorable for mass transfer, resulting in ready availability of catalytically active sites to substrate molecules and rapid release of the products in heterogeneous catalytic reaction.^20^ The surface composition and valence state of Pd–Cu@HO-NPC were examined by X-ray photoelectron spectroscopy (XPS) measurements. In the XPS survey spectrum (Fig. S5†), the binding energy peaks at around 284.1, 335.9, 399.7, 532.0 and 931.9 eV are assigned to C 1s, Pd 3d, N 1s, O 1s and Cu 2p, respectively.^44^ The deconvolution of the Pd 3d XPS spectrum presents two sets of double peaks corresponding to Pd 3d_5/2_ and Pd 3d_3/2_ (Fig. 4b). The binding energy peaks at 335.34 and 340.68 eV correspond to Pd(0) 3d_5/2_ and 3d_3/2_, respectively, while the peaks at 337.44 and 342.68 eV are attributed to Pd(ii) species. The ratio of surface Pd(0) to Pd(ii) is 1.26 as determined by the ratio of their relative peak areas. In the Cu 2p XPS spectrum, the two sets of double peaks are ascribed to Cu 2p_3/2_ and Cu 2p_1/2_ of Cu(0) and Cu(ii) species, respectively (Fig. 4c). The binding energy peaks at 932.18 and 957.23 eV are assigned to Cu(0) 2p_3/2_ and 2p_1/2_, respectively, while the peaks at 934.61 and 954.40 eV correspond to Cu(ii) 2p_3/2_ and 2p_1/2_, respectively. Impressively, the Pd(0) 3d_5/2_ and Cu(0) 2p_3/2_ peaks shift negatively by 0.06 and 0.11 eV, respectively, when compared with those in free Pd (335.4 eV) and Cu (932.29 eV).^45^ The negative shift probably results from the strong interaction between Pd–Cu alloy NPs and nitrogen-doped carbon, which makes Pd(0) and Cu(0) species more electron-rich than free Pd and Cu.^14^ In the high-resolution N 1s XPS spectrum (Fig. 4d), the presence of pyridinic N (398.5 eV), pyrrolic N (399.7 eV) and graphitic N (401.1 eV) could be clearly identified.^46^ The Raman spectrum shows the characteristic D-band and G-band at 1345 and 1577 cm^–1^, respectively (Fig. S6†). The relative ratio of *I*_D_/*I*_G_ is 0.98, indicating that the carbon species in Pd–Cu@HO-NPC possess a high graphitization degree, which is favorable for electron mobility and stabilization of the reactive intermediates in the radical-involved catalytic systems.^47^

The attractive advantages of the hollow octahedral nanostructure incorporated with well-dispersed Pd–Cu alloy NPs have encouraged us to explore its catalytic application. The selective oxidation of hydrocarbons is an essential reaction in organic synthesis and industrial chemistry; it has usually required the use of various oxidants, such as toxic metal oxides, peroxides and ozone.^48^ Compared with those oxidants, air is undoubtedly the most ubiquitous, atom-economical and environmentally benign oxidant. However, air is unreactive and difficult to activate, especially towards strong sp^3^ C–H bonds of hydrocarbons in the transformation. Recently, various supported noble metal nanocatalysts, such as palladium, platinum, ruthenium and gold, have been reported for selective oxidation of hydrocarbons, but the catalytic systems are subjected to high temperatures, high pressures and/or the use of the additives.^47^,^49^–^51^ Alloying a parent noble metal with a second metal offers numerous opportunities to improve catalytic activities, but the application of alloy metal NPs in this chemical transformation has seldom been reported.^52^

**Fig. 5:**
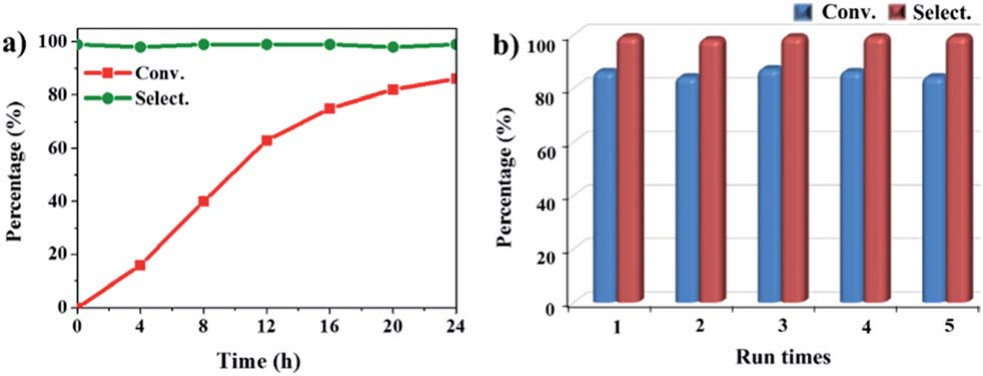
(a) The kinetic curve and (b) recyclability for Pd–Cu@HO-NPC in the oxidation reaction of indane. Reaction conditions: indane (8.77 mmol), catalyst ([Pd] = 0.2 mol%), air flow (5 mL min^–1^), 120 °C and 24 h.

The oxidation of indane was evaluated initially using air as an oxidant in the presence of 0.2 mol% palladium under solvent-free conditions. Kinetic profiles show that indane consumption and 1-indanone formation increased rapidly in the first 8 h, and 86% conversion of indane was achieved in 24 h (Fig. 5a). The selectivity of 1-indanone remains above 99% in the entire catalytic process. Control experiments were also performed. When the indane oxidation was carried out either in the absence of Pd–Cu@HO-NPC (Table 1, entry 2) or under a N_2_ atmosphere (entry 3), a negligible amount of product was detected after 24 h. As expected, the indane conversion decreased to 50% when hollow Pd–Cu@HO-NPC was replaced by dense Pd–Cu@NPC (entry 4). The use of the commercial Pd/C afforded a 33% conversion of indane under the same conditions (entry 5). The heterogeneous behavior of this catalytic system was verified by a hot filtration experiment. After the reaction was carried out for 6 h, the catalyst was quickly removed by filtration, and the reaction of the filtrate was continued for an additional 18 h, and both conversion and selectivity show negligible variation (entries 6 and 7).

**Table 1:** The aerobic oxidation of hydrocarbons catalysed by Pd–Cu@HO-NPC

Entry^*a*^	Substrate	Product	Conversion^*b*^ (%)	Selectivity^*b*^ (%)
1	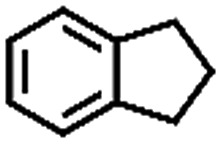	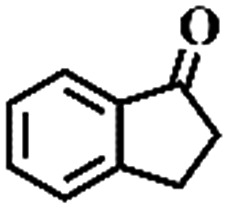	86	>99
2^*c*^	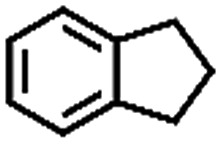	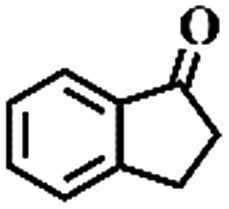	<1	—
3^*d*^	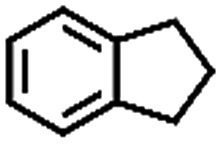	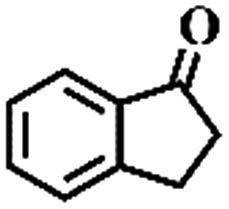	—	—
4^*e*^	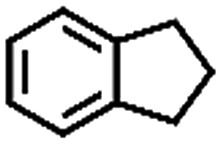	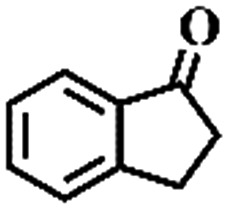	50	>99
5^*f*^	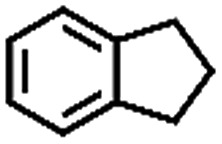	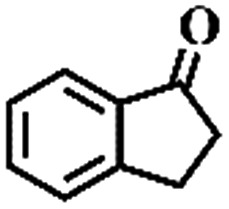	33	96
6^*g*^	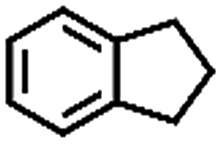	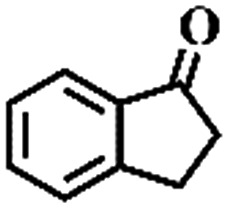	31	>99
7^*g*^	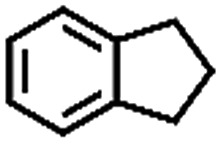	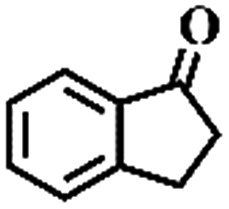	31	>99
8	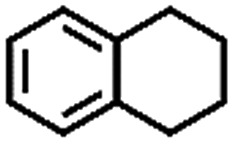	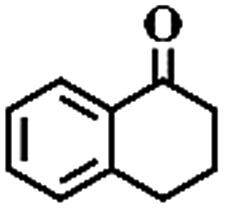	75	>97
9	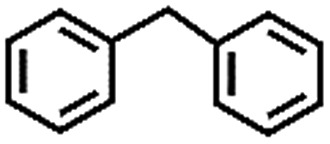	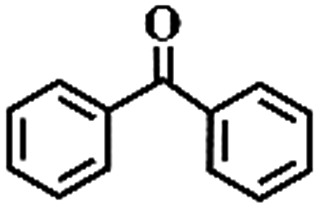	71	>99
10	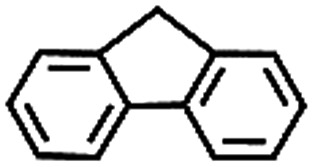	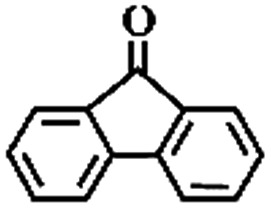	89	>99
11	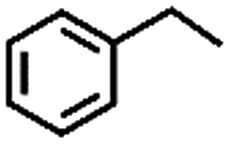	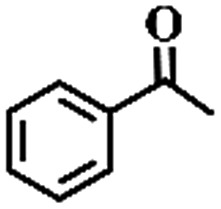	38	>99
12	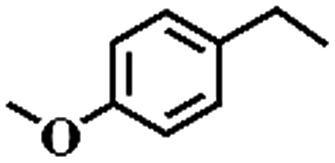	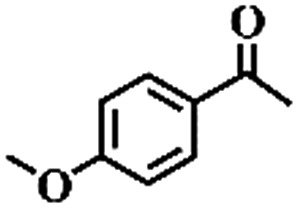	44	>93
13	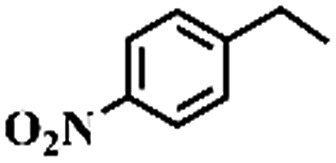	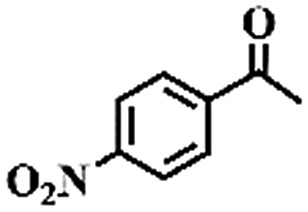	29	>95
14	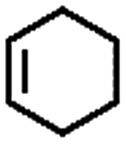	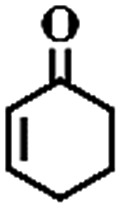	48	89
15^*h*^	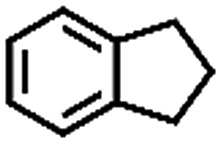	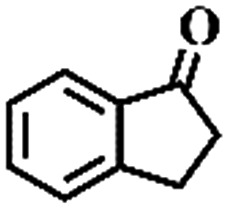	<0.3	—

^*a*^Reaction conditions: hydrocarbons (1 mL), catalyst ([Pd] = 0.2 mol%), air flow (5 mL min^–1^), 120 °C and 24 h.

^*b*^GC yield.

^*c*^The absence of catalysts.

^*d*^Pd–Cu@NPC was used as a catalyst.

^*e*^Under a N_2_ atmosphere.

^*f*^Pd/C was used as a catalyst.

^*g*^Filtration experiment.

^*h*^ *p*-Benzoquinone was added (1 mmol).

**Fig. 6:**
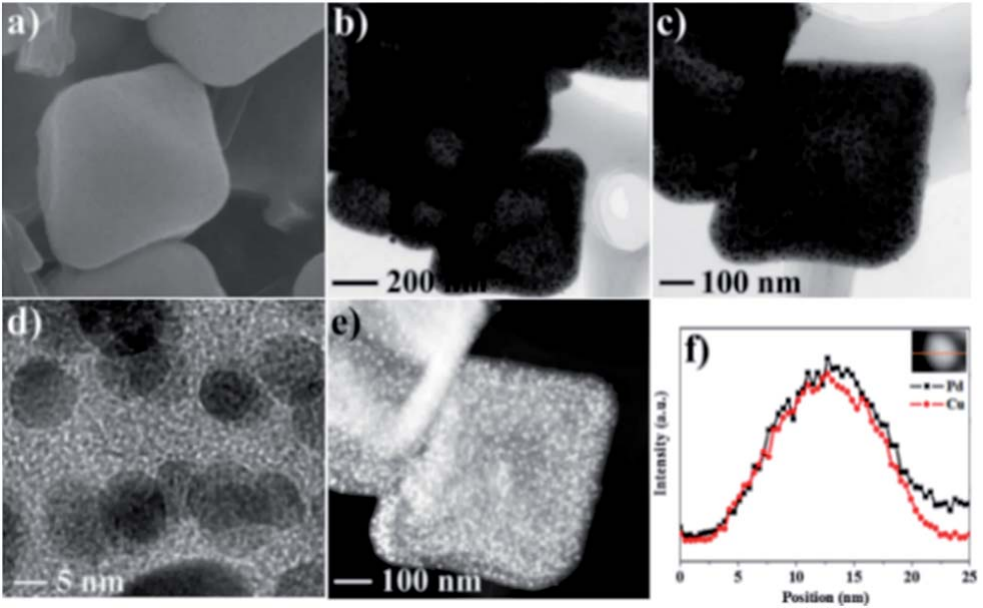
(a) SEM, (b–d) TEM, and (e) HAADF-STEM images and (f) the line-scan analysis results for Pd–Cu@HO-NPC-5run.

Pd–Cu@HO-NPC shows excellent recyclability and stability in the aerobic oxidation of indane. After the reaction finished, the catalyst was separated by centrifugation, and directly used for the next run. Pd–Cu@HO-NPC could be used for at least five runs without detectable loss of catalytic activity and selectivity (Fig. 5b), and the total turnover number (TON) exceeds 2125. The recovered Pd–Cu@HO-NPC after the consecutive reaction for five runs is denoted as Pd–Cu@HO-NPC-5run. As shown in Fig. 6 and S7,† the Raman spectrum, and SEM and TEM images of Pd–Cu@HO-NPC-5run are almost identical to those of the as-prepared sample. Most strikingly, Pd–Cu alloy NPs are still uniformly embedded in the hollow carbon material with the retention of their original size (Fig. S8†), and there are no metal NPs on the exterior of the porous carbon shell and/or in the hollow cavity. Inductively coupled plasma (ICP) analyses show that Pd and Cu contents in Pd–Cu@HO-NPC-5run are the same as those in the as-synthesized catalyst. These results demonstrate that hollow HO-NPC could effectively inhibit the leaching of catalytically active species and prevent the aggregation and migration of Pd–Cu alloy NPs in the hydrocarbon oxidation.

To extend the application scope of the catalytic system, the oxidation reactions of various hydrocarbons were examined using air as an oxidant. As shown in Table 1, a conversion of 75% was obtained in the oxidation of tetralin, a key intermediate in the commercial production of α-naphthol (entry 8).^48^,^52^ Diphenylmethane and fluorene with a large molecular size could be transformed into diphenylmethanone and fluorenone in good conversions of 71% and 89%, respectively (entries 9 and 10). Impressively, the C–H bond of ethylbenzene was also selectively oxidized, and acetophenone was obtained in a 38% GC yield (entry 11). The electron-rich 1-ethyl-4-methoxybenzene and electron-deficient 1-ethyl-4-nitrobenzene provided the corresponding products in 44% and 29% GC yields, respectively (entries 12 and 13), indicating that the electron-donating groups in ethylbenzene are more favorable for C–H bond activation than the electron-withdrawing groups in this catalytic system. Besides aromatic molecules, Pd–Cu@HO-NPC is also effective for the oxidation of aliphatic molecules. 48% conversion and 89% selectivity were achieved in 24 h when cyclohexene was used as a substrate (entry 14). These results show that Pd–Cu@HO-NPC is a promising catalyst for the aerobic oxidation of allylic or benzylic sp3 C–H bonds. Interestingly, when the oxidation of indane was tested in the presence of *p*-benzoquinone, which is a typical radical scavenger, the reaction was totally suppressed to give a negligible conversion (entry 15), indicating that the reaction proceeds through radical intermediates. The attractive catalytic performance of Pd–Cu@HO-NPC in selective aerobic oxidation of hydrocarbons is mainly attributed to its favorable structural features: (1) the hollow cavity and mesoporous shell are beneficial for mass transfer/diffusion of substrates and products; (2) the uniform distribution of Pd–Cu alloy NPs in the mesoporous shell makes them readily interact with hydrocarbons and oxygen molecules; (3) the incorporation of nitrogen atoms into the carbon shell not only makes Pd–Cu alloy NPs electron-rich, but also significantly enhances the interactions between the carbon shell and the embedded metal NPs; and (4) the high graphitization of the carbon shell benefits the electron mobility and radical stabilization during catalytic oxidation of hydrocarbons.

**Fig. 7:**
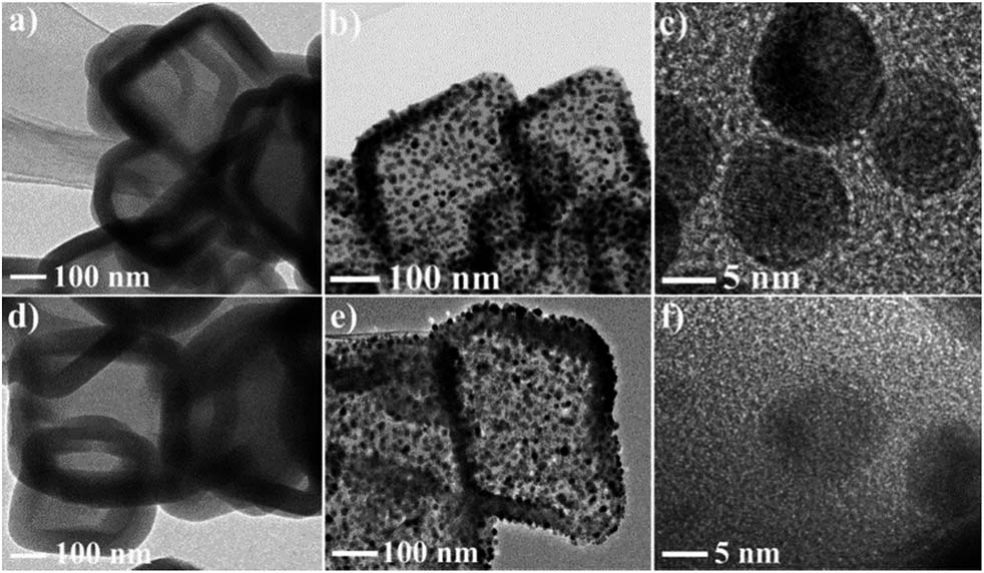
TEM images of (a) Pt–Cu@HO-ImIP, (b and c) Pt–Cu@HO-NPC, (d) Pd–Pt–Cu@HO-ImIP and (e and f) Pd–Pt–Cu@HO-NPC.

This strategy for the synthesis of Pd–Cu@HO-NPC exhibits excellent generality, and can be extended to the fabrication of other Cu-based bimetallic and trimetallic alloy NPs. The treatment of HKUST-1@ImIP with aqueous K_2_PtCl_6_ and Na_2_PdCl_4_/K_2_PtCl_6_ solutions generated hollow Pt–Cu@HO-ImIP and Pd–Pd–Cu@HO-ImIP, respectively (Fig. 7a and d). Their morphology and size are almost identical to those of Pd–Cu@HO-ImIP, suggesting that the change of exchanged metal ions has no obvious effects on the flexible ImIP shells. Notably, the pyrolysis of Pt–Cu@HO-ImIP and Pd–Pd–Cu@HO-ImIP under an inert atmosphere gave rise to the corresponding bimetallic Pt–Cu and trimetallic Pd–Pt–Cu alloy NPs supported by hollow nitrogen-doped carbon shells. TEM analyses indicate that Pt–Cu@HO-NPC and Pd–Pt–Cu@HO-NPC also possess hollow octahedral morphology, and the edge lengths and shell thicknesses are in the range of 200–500 nm and 30–60 nm, respectively (Fig. 7). The alloy NPs are tightly embedded in the HO-NPC shell, and their size, location and distribution are very similar to those in Pd–Cu@HO-NPC.

## Conclusions

HKUST-1@ImIP core–shell materials have been fabricated and employed as promising precursors of Cu-based alloy NPs embedded in hollow nitrogen-doped porous carbon. HKUST-1 is simultaneously decomposed when metal-containing anions are introduced into the ImIP shell through anion exchange in water, no tedious steps or hazardous reagents are required for the removal of templates, and the morphology and size of the flexible shell are well maintained. To the best of our knowledge, this is the first report on hollow polymers with flexible backbones. The released Cu ions and exchanged metal ions reside in the pores of the ImIP shell; subsequent topotactic transformation generates hollow nitrogen-doped porous carbon incorporated with Cu-based bimetallic or trimetallic alloy NPs. The unique hollow structure endows metal alloy NPs with high catalytic activity and outstanding durability in selective aerobic oxidation of hydrocarbons. In summary, this study develops a simple, green and general method for facile synthesis of Cu-based alloy NPs supported by hollow carbon materials. The synthetic strategy holds great promise for the synthesis of a broad range of hollow carbon nanostructures owing to the availability of a large family of MOFs and ionic polymers. Further investigation of other metal alloy NPs supported by various hollow heteroatom-doped carbon materials is in progress.

## Conflicts of interest

There are no conflicts to declare.

## Supplementary Material

Supplementary information

## References

[cit1] Wang G. H., Hilgert J., Richter F. H., Wang F., Bongard H. J., Spliethoff B., Weidenthaler C., Schüth F. (2014). Nat. Mater..

[cit2] Li X. H., Antonietti M. (2013). Chem. Soc. Rev..

[cit3] Liu L., Corma A. (2018). Chem. Rev..

[cit4] Li S., Tuel A., Laprune D., Meunier F., Farrusseng D. (2015). Chem. Mater..

[cit5] Yang H., Bradley S. J., Chan A., Waterhouse G. I. N., Nann T., Kruger P. E., Telfer S. G. (2016). J. Am. Chem. Soc..

[cit6] Chen L., Zhang L., Chen Z., Liu H., Luque R., Li Y. (2016). Chem. Sci..

[cit7] Li B., Nam H., Zhao J., Chang J., Lingappan N., Yao F., Lee T. H., Lee Y. H. (2017). Adv. Mater..

[cit8] Zhang W., Jiang X., Zhao Y., Carne-Sanchez A., Malgras V., Kim J., Kim J. H., Wang S., Liu J., Jiang J. S., Yamauchi Y., Hu M. (2017). Chem. Sci..

[cit9] Liu J., Wickramaratne N. P., Qiao S. Z., Jaroniec M. (2015). Nat. Mater..

[cit10] Chen C., Wu A., Yan H., Xiao Y., Tian C., Fu H. (2018). Chem. Sci..

[cit11] Luo W., Sankar M., Beale A. M., He Q., Kiely C. J., Bruijnincx P. C. A., Weckhuysen B. M. (2015). Nat. Commun..

[cit12] Pan H., Cheng Z., Xiao Z., Li X., Wang R. (2017). Adv. Funct. Mater..

[cit13] Yang J., Zhang F., Lu H., Hong X., Jiang H., Wu Y., Li Y. (2015). Angew. Chem., Int. Ed..

[cit14] He L., Weniger F., Neumann H., Beller M. (2016). Angew. Chem., Int. Ed..

[cit15] Jia R., Chen J., Zhao J., Zheng J., Song C., Li L., Zhu Z. (2010). J. Mater. Chem..

[cit16] Zhu Q. L., Li J., Xu Q. (2013). J. Am. Chem. Soc..

[cit17] Guo L. T., Cai Y. Y., Ge J. M., Zhang Y. N., Gong L. H., Li X. H., Wang K. X., Ren Q. Z., Su J., Chen J. S. (2015). ACS Catal..

[cit18] Tang J., Liu J., Salunkhe R. R., Wang T., Yamauchi Y. (2016). Chem. Commun..

[cit19] Wang Y., Yu L., Lou X. W. (2016). Angew. Chem., Int. Ed..

[cit20] Yang S., Peng L., Huang P., Wang X., Sun Y., Cao C., Song W. (2016). Angew. Chem., Int. Ed..

[cit21] Yang S., Zhu Y., Cao C., Peng L., Li S., Zhai D., Song W. (2017). Nanoscale.

[cit22] Gong Y., Zhong H., Liu W., Zhang B., Hu S., Wang R. (2018). ACS Appl. Mater. Interfaces.

[cit23] Xin B., Hao J. (2014). Chem. Soc. Rev..

[cit24] Xin B., Jia C., Li X. (2016). Curr. Org. Chem..

[cit25] Zhang L., Wu H. B., Lou X. W. (2013). J. Am. Chem. Soc..

[cit26] Chun J., Kang S., Park N., Park E. J., Jin X., Kim K. D., Seo H. O., Lee S. M., Kim H. J., Kwon W. H., Park Y. K., Kim J. M., Kim Y. D., Son S. U. (2014). J. Am. Chem. Soc..

[cit27] Zou F., Chen Y. M., Liu K., Yu Z., Liang W., Bhaway S. M., Gao M., Zhu Y. (2016). ACS Nano.

[cit28] Shao J., Wan Z., Liu H., Zheng H., Gao T., Shen M., Qu Q., Zheng H. (2014). J. Mater. Chem. A.

[cit29] Zhang X., Qin W., Li D., Yan D., Hu B., Sun Z., Pan L. (2015). Chem. Commun..

[cit30] Yang J., Zhang F., Lu H., Hong X., Jiang H., Wu Y., Li Y. (2015). Angew. Chem., Int. Ed..

[cit31] Guo P., Dutta D., Wong-Foy A. G., Gidley D. W. (2015). J. Am. Chem. Soc..

[cit32] Hasan Z., Jhung S. H. (2015). J. Hazard. Mater..

[cit33] Zhang S., Liu H., Liu P., Yang Z., Feng X., Huo F., Lu X. (2015). Nanoscale.

[cit34] Yu C., Cui J., Wang Y., Zheng H., Zhang J., Shu X., Liu J., Zhang Y., Wu Y. (2018). Appl. Surf. Sci..

[cit35] Zhuang J. L., Ceglarek D., Pethuraj S., Terfort A. (2011). Adv. Funct. Mater..

[cit36] Zhao H., Li X., Li L., Wang R. (2015). Small.

[cit37] Zhong H., Liu C., Wang Y., Wang R., Hong M. (2016). Chem. Sci..

[cit38] Jang J. S., Koo W. T., Choi S. J., Kim I. D. (2017). J. Am. Chem. Soc..

[cit39] Tang J., Salunkhe R. R., Liu J., Torad N. L., Imura M., Furukawa S., Yamauchi Y. (2015). J. Am. Chem. Soc..

[cit40] Dong Z. P., Le X. D., Liu Y. S., Dong C. X., Ma J. (2014). J. Mater. Chem. A.

[cit41] Mori K., Tanaka H., Dojo M., Yoshizawa K., Yamashita H. (2015). Chem.–Eur. J..

[cit42] Yuan M., Liu A., Zhao M., Dong W., Zhao T., Wang J., Tang W. (2014). Sens. Actuators, B.

[cit43] Yang F., Zhang Y., Liu P. F., Cui Y., Ge X. R., Jing Q. S. (2016). Int. J. Hydrogen Energy.

[cit44] Hronec M., Fulajtárová K., Vávra I., Soták T., Dobrocka E., Micusík M. (2016). Appl. Catal., B.

[cit45] Du X., Luo S., Du H., Tang M., Huang X., Shen P. K. (2016). J. Mater. Chem. A.

[cit46] Li L., Li L., Cui C., Fan H., Wang R. (2017). ChemSusChem.

[cit47] Lin X., Nie Z., Zhang L., Mei S., Chen Y., Zhang B., Zhu R., Liu Z. (2017). Green Chem..

[cit48] Zhang P., Gong Y., Li H., Chen Z., Wang Y. (2013). Nat. Commun..

[cit49] Maegawa T., Akashi A., Yaguchi K., Iwasaki Y., Shigetsura M., Monguchi Y., Sajiki H. (2009). Chem.–Eur. J..

[cit50] Kesavan L., Tiruvalam R., Rahim M. H. A., Saiman M. I. B., Enache D. I., Jenkins R. L., Dimitratos N., Lopez-Sanchez J. A., Taylor S. H., Knight D. W., Kiely C. J., Hutchings G. J. (2011). Science.

[cit51] Deng D., Yang Y., Gong Y., Li Y., Xu X., Wang Y. (2013). Green Chem..

[cit52] Takahashi M., Koizumi H., Chun W. J., Kori M., Imaoka T., Yamamoto K. (2017). Sci. Adv..

