# Supplementary material for: Facile fabrication of Cu-based alloy nanoparticles encapsulated within hollow octahedral N-doped porous carbon for selective oxidation of hydrocarbons
Source: Chem Sci. 2018 Sep 18;9(46):8703–10. doi: 10.1039/c8sc03531h (PMC6263394; doi:10.1039/c8sc03531h)
Supplement: Supplementary file 1 [file SC-009-C8SC03531H-s001.pdf]

Electronic Supplementary Material (ESI) for Chemical Science.
This journal is © The Royal Society of Chemistry 2018

Supporting information

for

Facile fabrication of Cu-based alloy nanoparticles encapsulated within hollow octahedral N-doped porous carbon for selective oxidation of hydrocarbons

Hong Zhong,[ab] Yangxin Wang,[a] Caiyan Cui,[a] Feng Zhou,[a] Shuangqi Hu[b] and Ruihu Wang*[a]

[a] State Key Laboratory of Structural Chemistry, Fujian Institute of Research on the Structure of Matter, Chinese Academy of Sciences,Fuzhou, Fujian 350002, China

[b] School of Environmental and Safety Engineering, North University of China, Taiyuan 030051, China

*Corresponding authors. E-mail: ruihu@fjirsm.ac.cn

Experimental section

General Information

HKUST-1 [S1] and 1,3,5-tri(1H-imidazol-1-yl)benzene [S2] were prepared according to literature methods. Other chemicals were commercially available and used without further purification. FTIR spectra were recorded with KBr pellets using Perkin-Elmer instrument. Thermal gravimetric analysis (TGA) was carried out on NETZSCH STA

449C by heating samples from 30 to 800 ^{o}C in a dynamic nitrogen atmosphere with a heating rate of 10 $^{o}C \cdot min^{-1}$. Powder X-ray diffraction (PXRD) patterns were recorded in the range of 2θ = 5-85 ^{o} on a desktop X-ray diffractometer (RIGAKU-Miniflex II) with Cu Kα radiation (λ = 1.5406 Å). Nitrogen adsorption and desorption isotherms were measured at 77 K using a Micromeritics ASAP 2020 system. The samples were degassed at 120 ^{o}C for 10 h before the measurements. Surface areas were calculated from the adsorption data using Brunauer-Emmett-Teller (BET) methods. The pore size distribution curves were obtained from the adsorption branches using non-local density functional theory (NLDFT) method. Field-emission scanning electron microscopy (SEM) was performed on a JEOL JSM-7500F operated at an accelerating voltage of 3.0 kV. Raman spectra were obtained using a Renishaw UV-1000 Photon Design spectrometer at 532 nm excitation focused through a 100 × microscope objective for a total interrogation spot size of ~1 μm. Transmission electron microscope (TEM) images were obtained with a JEOL JEM-2010 instrument operated at 200 kV. X-ray photoelectron spectroscopy (XPS) measurements were performed on a Thermo ESCALAB 250 spectrometer, using non-monochromatic Al Kα X-ray as the excitation source and choosing C 1s (284.6 eV) as the reference line. Inductively coupled plasma spectroscopy (ICP) was measured on Jobin Yvon Ultima2. Gas chromatography (GC) was performed on a Shimadzu GC-2014 equipped with a capillary column (RTX-5, 30 m×0.25 μm) using a flame ionization detector.

Synthesis of HKUST-1@ImIP: A mixture of HKUST-1 (200 mg) and 1,2,4,5-tetrakis(bromomethyl)benzene (0.25 mmol, 112 mg) in MeCN (40 mL) and DMF (10 mL) was ultrasonically dispersed for 0.5 h. Then 1,3,5-tri(1H-imidazol-1-yl)benzene

(0.33 mmol, 92 mg) in MeCN (40 mL) and DMF (10 mL) was added and stirred at 85 ^{o}C for 5 h. The resultant precipitate was collected by centrifugation, washed with MeCN (3 x 30 mL) and dried *in vacuo* to give rise to HKUST-1@ImIP. Yield: 384 mg (96%). Elemental analysis: C, 52.32; H, 4.21; N, 10.36. FTIR (KBr cm^{-1}): 3429 (s), 3083 (w), 1621 (s), 1550 (m), 1506 (w), 1448 (w), 1376 (s), 1191 (w), 1105(w), 1008 (w), 758 (w), 722 (w), 666 (w).

Synthesis of Pd-Cu@HO-ImIP: HKUST-1@ImIP (400 mg) was added into an aqueous solution (50 mL) of Na_2PdCl_4 (0.50 mol, 147 mg), the mixture was stirred vigorously at room temperature for 24 h. The resultant product was washed thoroughly with copious H_2O, and dried *in vacuo* at 80 ^{o}C for 12 h to give rise to Pd-Cu@HO-ImIP. Yield: 376 mg (85 %). Elemental analysis: C, 55.76; H, 3.63; N, 14.82. FTIR (KBr cm^{-1}): 3603 (w), 3399 (w), 3133 (m), 3067 (w), 1708 (m), 1616 (s), 1550 (s), 1442 (m), 1375 (m), 1227 (m), 1187 (m), 1095 (m), 737 (m), 676 (w), 625 (w).

Synthesis of Pd-Cu@HO-NPC: Pd-Cu@HO-ImIP (300 mg) in a ceramic boat was heated to 500 ^{o}C in a heating rate of 5 ^{o}C min^{-1} under N_2 atmosphere, and then kept at this temperature for 2 h. After cooled to room temperature, the resultant black powders were treated with 10 M aqueous KOH solution at 100 ^{o}C for 24 h, followed by washing with copious water and methanol, and dried at 80 ^{o}C *in vacuo* overnight to afford Pd-Cu@HO-NPC.

Synthesis of Pt-Cu@HO-NPC: HKUST-1@ImIP (400 mg) was added into an aqueous solution (50 mL) of K_2PtCl_6 (0.50 mol, 243 mg), the mixture was stirred vigorously at room temperature for 24 h. The resultant solid was washed thoroughly with copious H_2O

and dried *in vacuo* at 80 °C for 12 h. The obtained powders were put into a ceramic boat and heated to 800 °C in a heating rate of 5 °C min^{-1} under N_2 atmosphere. After kept at this pyrolysis temperature for 2 h and cooled to room temperature, the resultant black powders were treated with 10 M aqueous KOH solution at 100 °C for 24 h, followed by washing with water and methanol, and dried at 80 °C *in vacuo* overnight to afford Pt-Cu@HO-NPC.

Synthesis of Pd-Pt-Cu@HO-NPC: HKUST-1@ImIP (400 mg) was added into an aqueous solution (50 mL) of Na_2PdCl_4 (0.25 mol, 73.5 mg) and K_2PtCl_6 (0.25 mol, 121.6 mg) the mixture was stirred vigorously at room temperature for 24 h. The resultant solid was washed thoroughly with copious H_2O and dried *in vacuo* at 80 °C for 12 h. The obtained powders were put into a ceramic boat and heated to 600 °C in a heating rate of 5 °C min^{-1} under N_2 atmosphere. After kept at this pyrolysis temperature for 2 h and cooled to room temperature, the resultant black powders were treated with 10 M aqueous KOH solution at 100 °C for 24 h, followed by washing with water and methanol, and dried at 80 °C *in vacuo* overnight to afford Pd-Pt-Cu@HO-NPC.

Synthesis of Pd-Cu@NPC: HKUST-1 (200 mg) was dissolved into an aqueous solution (50 mL) of Na_2PdCl_4 (0.50 mol, 147 mg), then ImIP (400 mg) was added into the mixture and stirred vigorously at room temperature for 24 h. The resultant solid was washed thoroughly with copious H_2O and dried *in vacuo* at 80 °C for 12 h. The obtained powders were put into a ceramic boat and heated to 600 °C in a heating rate of 5 °C min^{-1} under N_2 atmosphere. After kept at this pyrolysis temperature for 2 h and cooled to room temperature, the resultant black powders were treated with 10 M aqueous KOH solution

at 100 ^{o}C for 24 h, followed by washing with water and methanol, and dried at 80 ^{o}C *in vacuo* overnight to afford Pd-Cu@NPC.

General procedures for oxidation of hydrocarbons with air: Oxidation was carried out in a high-pressure reactor equipped with a magnetic stirrer and an air inlet tube. The mixture of substrate (1 mL) and catalysts (0.2 mmol%) was stirred at indicated temperature. A stream of air produced by air pump was conducted into the reaction mixture and controlled by a constant flow rate (5 mL min^{-1}). After the reaction was completed, the product was removed from the reaction mixture with syringe. Conversion and selectivity were determined by GC.

General procedures for recyclability test: After oxidation reaction of indane in the presence of Pd-Cu@HO-NPC under air flow at 120 °C was finished, the product was removed directly from the reaction mixture with syringe, conversion and selectivity were determined by GC. The black solid was washed with ethanol, the recovered catalytic species were dried *in vacuo* and reused for the next run with the recharge of fresh indane.

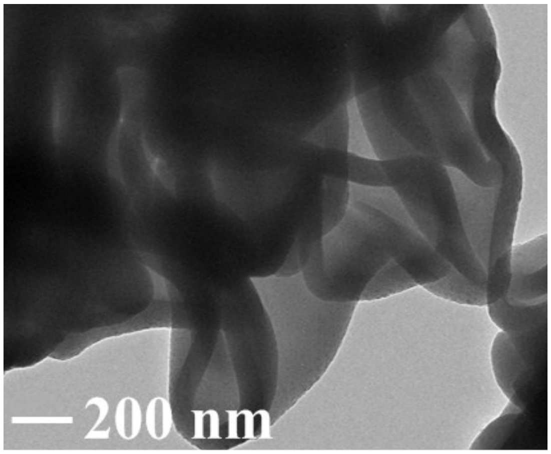

Figure S1. TEM image of Pd-Cu@HO-ImIP after washed with aqueous EDTA-2Na solution to remove metal ions.

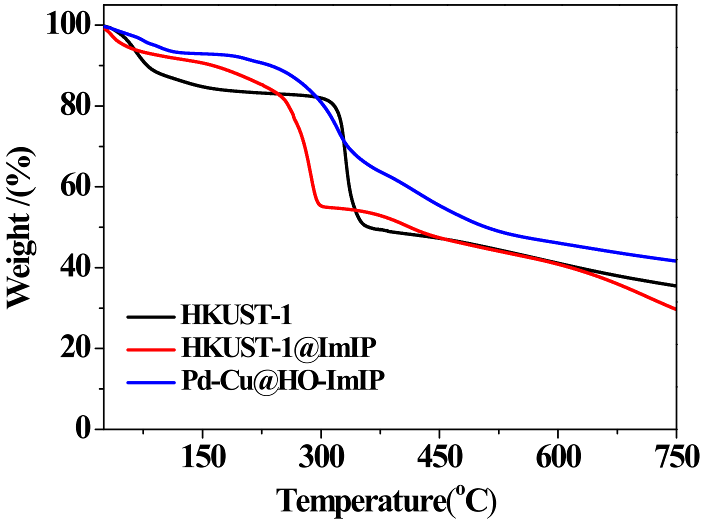

Figure S2. TGA curves of HKUST-1, ImIP, HKUST-1@ImIP and Pd-Cu@HO-ImIP.

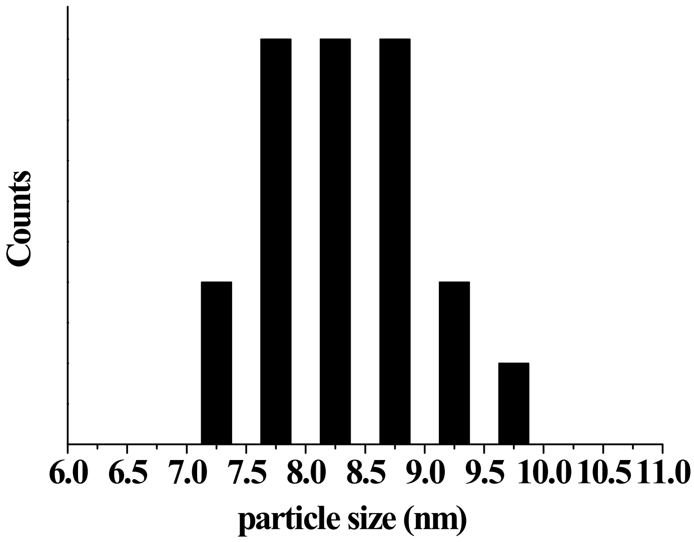

Figure S3. The size distribution of Pd-Cu alloy NPs in Pd-Cu@HO-NPC.

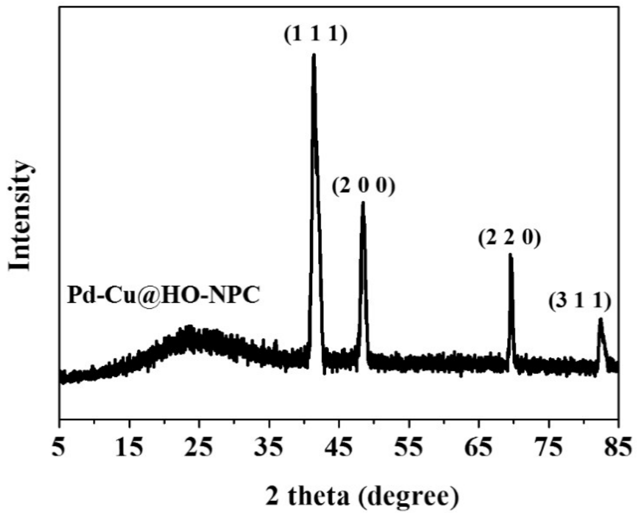

Figure S4. XRD pattern of Pd-Cu@HO-NPC.

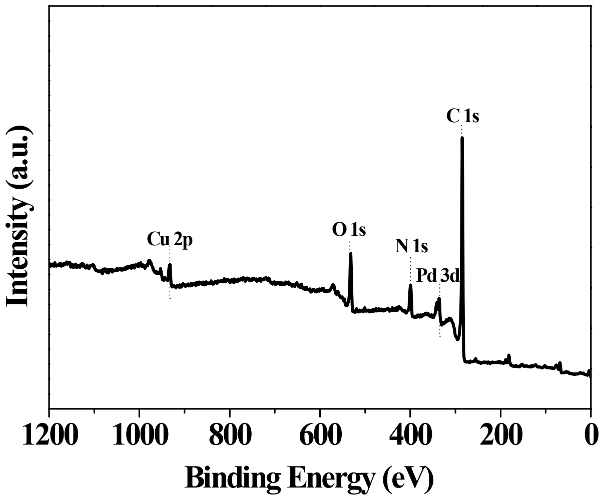

Figure S5. XPS survey spectrum of Pd-Cu@HO-NPC.

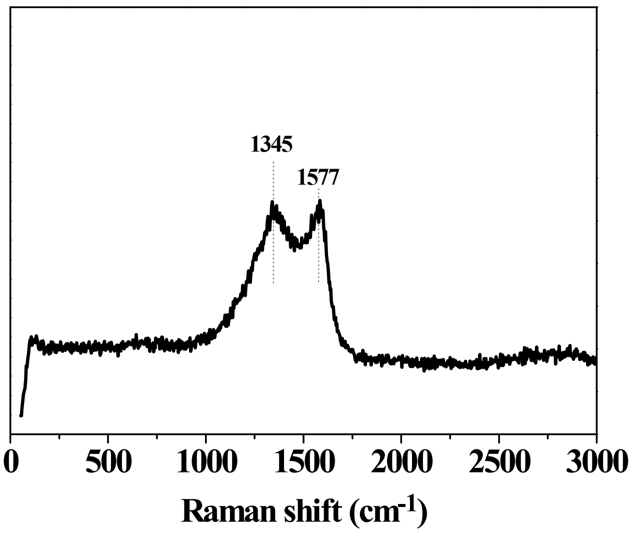

Figure S6. Raman spectrum of Pd-Cu@HO-NPC.

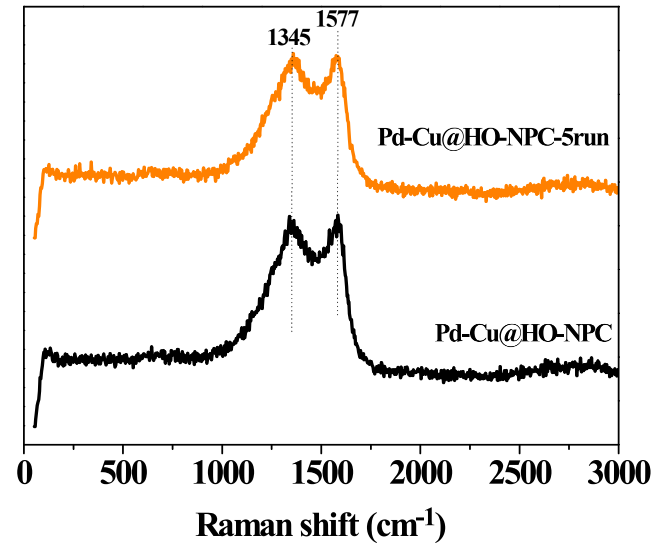

Figure S7. The Raman spectrum of Pd-Cu@HO-NPC-5run.

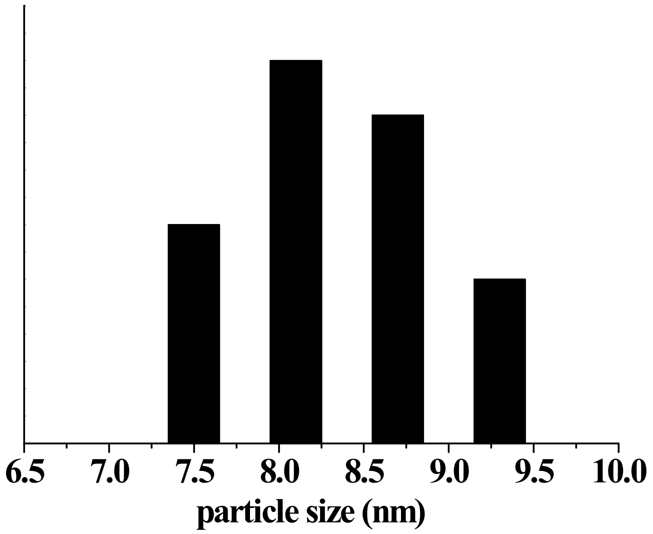

Figure S8. The size distribution of Pd-Cu alloy NPs in Pd-Cu@HO-NPC-5run

Reference

[S1] J. L. Zhuang, D. Ceglarek, S. Pethuraj and A. Terfort, *Adv. Funct. Mater.*, 2011, **21**, 1442-1447.

[S2] A. Rit, T. Pape and F. E. Hahn, *J. Am. Chem. Soc.,* 2010, **132**, 4572-4573.
